# In vitro polymyxin activity against clinical multidrug-resistant fungi

**DOI:** 10.1186/s13756-019-0521-7

**Published:** 2019-04-24

**Authors:** Hanane Yousfi, Stéphane Ranque, Jean-Marc Rolain, Fadi Bittar

**Affiliations:** 1 Aix Marseille Univ, IRD, APHM, MEPHI, IHU Méditerranée Infection, 19-21 boulevard Jean Moulin, 13005 Marseille, France; 2 Aix Marseille Univ, IRD, APHM, SSA, VITROME, IHU-Méditerranée Infection, 19-21 boulevard Jean Moulin, 13005 Marseille, France

**Keywords:** Polymyxin antibiotics, MDR-fungi, Repurposing-drug, *Candida albicans*, Molds

## Abstract

**Background:**

Although antifungals are available and usually used against fungal infections, multidrug-resistant (MDR) fungal pathogens are a growing problem for public health. Moreover, fungal infections have become more prevalent nowadays due to the increasing number of people living with immunodeficiency. Thus, previously rarely-isolated and/or unidentified fungal species including MDR yeast and moulds have emerged around the world. Recent works indicate that polymyxin antibiotics (polymyxin B and colistin) have potential antifungal proprieties. Therefore, investigating the in vitro activity of these molecules against clinical multidrug-resistant yeast and moulds could be very useful.

**Methods:**

In this study, a total of 11 MDR yeast and filamentous fungal strains commonly reported in clinical settings were tested against polymyxin antibiotics. These include strains belonging to the *Candida*, *Cryptococcus* and *Rhodotorula* yeast genera, along with others belonging to the *Aspergillus*, *Fusarium*, *Scedosporium*, *Lichtheimia* and *Rhizopus* mould genera. The fungicidal or fungistatic action of colistin against clinical yeast strains was determined by the time-kill study. Further, a checkerboard assay for its combination with antifungal agents, usually used in clinical practices (amphotericin B, itraconazole, voriconazole), was carried out against multi-drug resistant fungal strains.

**Results:**

Polymyxin B and colistin exhibited an antifungal activity against all MDR fungal strains tested with MICs ranging from 16 to 128 μg/ml, except for the *Aspergillus* species. In addition, colistin has a fungicidal action against yeast species, with minimum fungicidal concentrations ranging from 2 to 4 times MICs. It induces damage to the MDR *Candida albicans* membrane. A synergistic activity of colistin-amphotericin B and colistin-itraconazole associations against *Candida albicans* and *Lichtheimia corymbifera* strains, respectively, and colistin-fluconazole association against *Rhodotorula mucilaginosa,* was demonstrated using a checkerboard microdilution assay.

**Conclusion:**

colistin could be proposed, in clinical practice, in association with other antifungals, to treat life-threatening fungal infections caused by MDR yeasts or moulds.

## Background

Invasive fungal diseases treatment is challenged by the restricted number of available antifungal drugs; with only four different classes of antifungals being available to treat a large number of fungal-associated diseases [1]. (i) Polyenes are the first antifungals available in clinical practice with two drugs mainly used: amphotericin B and nystatin. (ii) Azole antifungals, such as fluconazole, itraconazole, voriconazole, posaconazole and isavuconazole, are the most common drugs used in different clinical situations; azole drugs display a large spectrum of activity against both yeast and filamentous fungi. (iii) Pyrimidine analogues, including 5-flucytosine, are used in combination with other antifungals to treat yeast infections but have little action against most moulds. (iv) Finally, echinocandins, the newest class of antifungals include caspofungin, micafungin and anidulafungin that display fungicidal activity against ascomycetes yeast species [2].

In addition to this limited therapeutic arsenal, there has been a dramatic and worldwide increase in the incidence of fungal infections [3]. In fact, along with the main mycosis agents, such as *Candida albicans*, *Aspergillus fumigatus* and *Cryptococcus neoformans* [4], other life-threatening and emerging pathogens, including not previously well-identified/characterized species and opportunistic multidrug-resistant (MDR) ones, are increasingly reported. These include *Candida auris*, *Scedosporium/Lomentospora* spp.*, Fusarium* spp. and Mucorales [2]. Indeed, several factors can explain this increasing incidence of fungal infections; the increasing number of patients with immunodeficiency (ex. HIV, cancer and transplant patients), the ageing of the population [5] and improved detection and diagnostic methods [2]. However, the severity of such fungal infections varies depending on the site of infection (superficial or deep-seated) and the immune status of the concerned patients. One major characteristic of these emerging fungal pathogens is their highly-resistant profile to antifungal drugs. Therefore, these disseminated infections caused by MDR yeasts and moulds are difficult to treat [6], leading thus to a significant increase of morbidity and mortality, in immunocompromised patients but also in healthy individuals [7]. Antifungal resistance can be intrinsic, called primary resistance, or acquired, also called secondary resistance. Many resistance mechanisms have been described, such as biofilm formation (especially in *Candida albicans*), failure of intracellular drug accumulation or drug target alterations [8]. During the past decade, genome plasticity of human fungal pathogens has been strongly associated with their ability to acquire resistance to antifungals [9]. That is why many studies suggest to use other pharmacological classes and re-purposing old drugs either as a single antifungal agent or in combination with known antifungal drugs [10].

In this regard, antimicrobial peptides (AMPs) have received attention as prospective compounds for a further discovery of new antimycotics. More than 2700 antimicrobial peptides have been identified and the number is growing [11]. Among the AMPs commonly used in therapeutic practices, there are polymyxins which are cyclic, positively charged peptides, obtained naturally from Gram-positive bacteria, such as *Paenibacillus polymyxa*. Among polymyxin molecules described, two have been used in clinical settings: polymyxin B (PMB) and polymyxin E (colistin) [12]. This class of antibiotics has been discarded in the early 1980s because of their neuro- and nephro-toxicity. However, polymyxins were recently reintroduced in the antimicrobial therapy as a last option to treat infections caused by multidrug-resistant Gram-negative bacteria [13]. In addition to its antibacterial action, polymyxins were shown in the early 1970s to have antifungal activity against various *Candida* species, including *Candida tropicalis* with polymyxin E Minimum inhibitory concentrations (MICs) ranging from 30 to 75 μg/ml [14]. More recently, polymyxin sensitivity of life-threatening moulds, such as *Fusarium* and *Rhizopus* species, has been described [15, 16]. This study aimed to test the in vitro activity of polymyxin against the most common clinical MDR yeasts and moulds and to assess colistin activity, the mechanism of action and the synergy of colistin-antifungals associations that could be used in the treatment of invasive fungal infections.

## Methods

### Fungal isolates

In this study, eleven clinical fungi recovered at the University hospital of Marseille were used. Four yeasts belonging to *Candida*, *Cryptococcus* and *Rhodotorula* species and seven moulds belonging to *Fusarium*, *Scedosporium*, *Lichtheimia*, *Rhizopus* and *Aspergillus* species were tested (Table 1). Isolates were identified using Matrix-Assisted Laser Desorption/Ionisation mass spectrometry (MALDI-TOF MS) [17] and microscopic methods for the filamentous fungi species. These strains were isolated from different clinical samples including blood culture, cerebrospinal fluid, nails, bronchial aspiration and ocular samples (Table 1). *Candida krusei* ATCC 6258, *Candida parapsilosis* ATCC 22019, *Aspergillus fumigatus* ATCC 204205, *Aspergillus flavus* ATCC 204304, *Escherichia coli* LH1 [18], *Escherichia coli* 1R4 [19] and *Klebsiella pneumoniae* 853 [20] were used as susceptibility testing quality controls.

**Table 1:** Phenotypic profiles and Colistin, PMB MICs of fungal strains tested in this study

	Clinical samples	MICs (μg/mL)											
		Anid	Mica	Caspo	Flu	5-Fc	Posa	Itra	Vorico	AB	Isavu	Ct	PMB
*Candida krusei* ATCC 6258	/	0.06	0.12	0.25	32	8	0.25	0.12	0.25	1	ND	64	32
*Candida parapsilosis* ATCC 22019	/	0.5	0.5	0.12	1	0.12	0.03	0.008	0.03	0.5	ND	64	16
*Rhodotorula mucilaginosa*	Endobucal	> 8	> 8	> 8	128	0.06	1	0.5	2	1	ND	32	16
*Cryptococcus neoformans*	Blood	> 8	> 8	> 8	2	1	0.03	0.12	0.03	0.5	ND	32	16
*Candida albicans* H5	CSF	0.015	0.03	0.05	16	1	0.5	0.5	0.25	0.25	ND	128	128
*Candida albicans* H6	Nails	0.06	0.03	0.12	> 256	0.12	> 8	32	256	1	ND	128	64
*Aspergillus fumigatus* ATCC 204205	/	ND	ND	ND	ND	ND	0.008	0.015	0.12	2	ND	> 256	> 256
*Aspergillus flavus* ATCC 204304	/	ND	ND	ND	ND	ND	0.06	0.06	1	4	ND	> 256	> 256
*Aspergillus calidoustus*	Bronchial aspiration	ND	ND	ND	ND	ND	1	6	4	4	0.25	256	128
*Fusarium oxysporum*Y5	Nails	ND	ND	ND	ND	ND	> 32	> 32	2	4	> 32	64	16
*Fusarium solani* Y6	Ocular sample	ND	ND	ND	ND	ND	> 32	> 32	> 32	> 32	> 32	64	16
*Rhizopus oryzae* Y9	Sinus biopsy	ND	ND	ND	ND	ND	> 32	> 32	> 32	> 32	> 32	128	64
*Lomentospora prolificans Y8*	Blood	ND	ND	ND	ND	ND	> 32	64	> 32	> 32	> 32	32	16
*Scedosporium apiospermum* F2	Bronchial aspiration	ND	ND	ND	ND	ND	2	0.75	> 32	4	ND	16	16
*Lichtheimia corymbifera* ST87	Eyes	ND	ND	ND	ND	ND	2	2	> 32	2	2	32	32

Anid - Anidulafungin; Mica - Micafungin; Casp - Caspofungin; Flu - Fluconazole; 5-Fc - 5-Flurocytosin; Pos - Posaconazol, Itra - Itraconazole; Vori - Voriconazole; AB - Amphotericin B; Isavu – Isavuconazole; Ct – Colistin; PMB- Polymyxin B; MIC - Minimum Inhibitory Concentration; ND: Not Done

### Phenotypic profiles determination

Antifungal susceptibility testing was performed using two different methods: E-test (BioMérieux, Marcy l’Etoile, France) and commercial broth microdilution plates; Sensititre®YeastOne® (Thermo Fisher Scientific, Schwerte, Germany). The MICs obtained for each antifungal tested against yeasts and moulds (Table 1) was compared to the breakpoints provided by the manufacturers or to the epidemiological cutoff as previously described [21] in order to assess the susceptibility of each strain to the different antifungal agents tested.

### Polymyxin susceptibility testing

Colistin and PMB MICs were performed using the broth microdilution method as outlined by the Clinical and Laboratory Standards Institute (CLSI) (M38-A, Vol. 22, NO. 16 for filamentous fungi and M27-A2, Vol. 22, NO. 15 for yeasts). Serial colistin and PMB (Sigma Aldrich, St Louis, France) dilutions ranging from 0.5 to 256 μg/ml were prepared in RPMI-1640 (Sigma Aldrich, St Louis, France) with glutamine and without bicarbonate medium buffered to pH 7.0 with MOPS (Sigma Aldrich, St Louis, France) buffer. Fungal inoculums were prepared in the test medium and adjusted to 0.5 MacFarland. A 1:100 dilution followed by a 1:20 dilution were performed on yeast strains to obtain a final inoculum of 0.5 to 2.5 × 10^3^ CFU/mL, whereas only a 1:50 dilution was done for moulds with a final inoculum of approximately 0.4 to 5 × 10^4^ CFU/mL. It is important to mention that fresh conidia of filamentous fungi were obtained after approximately 7 days of incubation at 35 °C on potato dextrose agar. Then, 100 μl of the fungal/bacterial inoculum was added into each colistin or PMB-containing wells. Plates were incubated at 37 °C for 24 h (*Candida* spp., bacterial strains) or 48 h (*Cryptococcus*) and at 35 °C for 48 h (filamentous fungi and *Rhodothorula mucilaginosa*). The susceptibility to polymyxin antibiotics was assessed on the basis of visual observation of growth or inhibition of the isolate in the culture media. The Resazurin (Sigma Aldrich, St Louis, France) was used to indicate the growth of any microorganism by a culture medium colour shift from blue to pink. Then, the inhibition rate was calculated, after measuring the optical density (OD) value by using a plate reader spectrophotometer (Multiskan spectrum, Thermo Scientific, France), as follows: % of fungal growth inhibition = (OD of untreated well - OD of tested well)*100 / (OD of untreated well – OD of blank well); where a blank well contains only the RPMI medium (i.e. without any fungal strain and without any antibiotic agent), an untreated well contains a given strain in the RPMI medium without any antibiotic agents and a tested well contains both a given strain in the RPMI medium and a given antibiotic agent. Thus, the growth inhibition rate is close to 100% when the concentration of an antibiotic reaches to the MIC as the OD of the tested well is quasi-equal to the OD of the blank well.

### Colistin time-kill experiment and minimum fungicidal concentration (MFC) determination

Colistin time-kill study was performed, as previously described [22]. 9 ml of the fungal suspension was adjusted to a 0.5 McFarland turbidity. One ml of the adjusted fungal suspension was added to 9 ml of either RPMI-1640 medium, as a control, or to a solution of growth medium supplemented with an appropriate concentration of antibiotic solution. The colistin concentrations in the resulting solutions were 0.5, 1, 2, 4, 8, 16, and 32 times the MICs for the tested isolates. Then, the tubes were incubated at 37 °C on an orbital shaker. At 0, 6, 12, 24, 36, and 48 h following the introduction of the tested isolate into the solutions tubes, 100 μl aliquots were taken from each test solution. Different serial dilutions were performed on these aliquots, and a 10 μl aliquot from each dilution was streaked on Sabouraud agar plates (Biomérieux, France) and incubated for approximately 24 h for colony count determination. Then, the Minimum fungicidal concentration (MFC) was determined as the lowest antibiotic concentration leading to no significant growth or less than three colonies on Sabouraud agar plates in comparison to the growth control. The experimental data was analyzed using the GraphPad Prism 5.3 software (GraphPad Inc., San Diego, CA, USA) to obtain time-kill curves.

### Study of the mechanism of action of colistin on *Candida albicans* species

We used propidium iodide (PI), a membrane-impermeable DNA stain, to demonstrate the eventual fungicidal activity of colistin by its ability to induce cell membrane damages [23]. Colistin-treated and untreated cells were suspended in PBS and stained with 5 μg/ml of PI for 20 min in the dark at room temperature. PI fluorescence was examined under a fluorescence microscope at λ_ex_ = 535 nm and λ_em_ = 617 nm.

### Colistin / antifungals association checkerboard testing

Checkerboard broth microdilution method was used to test the synergy of colistin with three antifungal agents commonly used in clinical settings; amphotericin B, fluconazole and itraconazole (Sigma Aldrich, St Louis, France). Firstly, MICs of individual agents were determined because the range of concentration of drugs to test the associations was established according to these MICs. Eight doubling dilutions of the two agents being tested (i.e. colistin and antifungals) were prepared in the susceptibility testing medium RPMI-1640 (1MIC, ½ MIC, ¼ MIC, 1/8 MIC, 1/16 MIC, 1/32 MIC, 1/64 MIC, 1/128 MIC). 50 μl of each agent was added in wells of a microtiter plate to provide a total of 64 drug combinations. Additional rows were used to determine the MIC of each antimicrobial agent alone by adding 100 ml of each agent. The fungal inoculum was prepared according to the CLSI standard protocol and 100 μl was added to each well. The plates were incubated under optimal growth conditions for yeasts and filamentous moulds. The results were analysed and MIC_100_ were determined visually and by optical density measurements on a microplates reader (Multiskan Spectrum, Thermo Scientific), based on a reduction in absorbance compared to the free drug control wells. Then, we calculated the Fractional Inhibitory Concentrations (FICs), where FIC1 (Colistin) = MIC of colistin in the combination/MIC of colistin alone and FIC2 (antifungal drug) = MIC of antifungal drug in the combination/MIC of antifungal drug alone. The fractional inhibitory index (FIX) is the sum of FIC1 and FIC2 and was interpreted as follows: if the FIX is ≤0.5, then there is synergy between the tested antimicrobials; if it is > 0.5 but ≤1 then there is additivity between the tested antimicrobials; if it is > 1 but ≤4, there is indifference between the tested antimicrobials, and if the FIX is > 4, that means that there is an antagonism between the tested antimicrobials.

## Results

### Resistance phenotypic profiles of the emerging fungal pathogens used in this study

The MICs obtained with the different antifungal classes tested against the 4 *Candida* spp. and *Aspergillus* spp. quality control strains corresponded to the recommended 24 h and 48 h - MICs limits of microbroth dilution method outlined in the CLSI protocol. This confirms the adequateness of the method used to determine susceptibility profiles for all isolates. *C. albicans* H6 was resistant to all azole antifungal agents tested while *C. albicans* H5 was only resistant to fluconazole (Table 1). Both strains remained sensitive to echinocandins and amphotericin B antifungals. *Rhodotorula mucilaginosa* was resistant in vitro to fluconazole with MIC = 128 μg/ml and to all echinocandin agents. *Cryptococcus neoformans* was also resistant to echinocandin class (Table 1).

All filamentous fungi tested were resistant to amphotericin B with a high MIC for *Rhizopus oryzae* and *Scedosporium* species (> 32 μg/ml). *Fusarium, Scedosporium* and *Rhizopus* species were also resistant to azoles (voriconazole, itraconazole and posaconazole) usually used in clinical settings (Table 1). The pyrimidine analogue 5-fluorocytosine and echinocandin agents were not tested against moulds because of their relatively poor activity against filamentous fungi.

### Polymyxins exhibited in vitro, antifungal activity against MDR yeasts and filamentous fungi

Susceptibility testing showed clear endpoints with a 100% growth inhibition of the MDR strains tested. The MICs of *Escherichia coli* LH1 (MIC = 8 μg/ml) (16), *Escherichia coli* 1R4 (MIC = 8 μg/ml) (17) and *Klebsiella pneumoniae* 853 (MIC = 64 μg/ml) [20] were within the quality control ranges.

The polymyxins MICs against the fungi strains ranged from 16 to 128 μg/ml (Table 1), except for *Aspergillus fumigatus* and *A. flavus* strains, which appeared to be not sensitive to both colistin and PMB, with MICs ≥256 μg/ml. Among the 11 clinical strains tested, 4 isolates including *Lichtheimia corymbifera*, *Lomentospora prolificans* and *Scedosporium apiospermum* exhibited the highest susceptibility to polymyxin molecules, with MICs ranging from 16 μg/ml to 32 μg/ml; in contrast *Aspergillus calidoustus* exhibited the lowest susceptibility to colistin and PMB, with MICs of 256 μg/ml and 128 μg/ml, respectively.

### Colistin presents fungicidal activity against clinical MDR yeasts

**Fig. 1:**
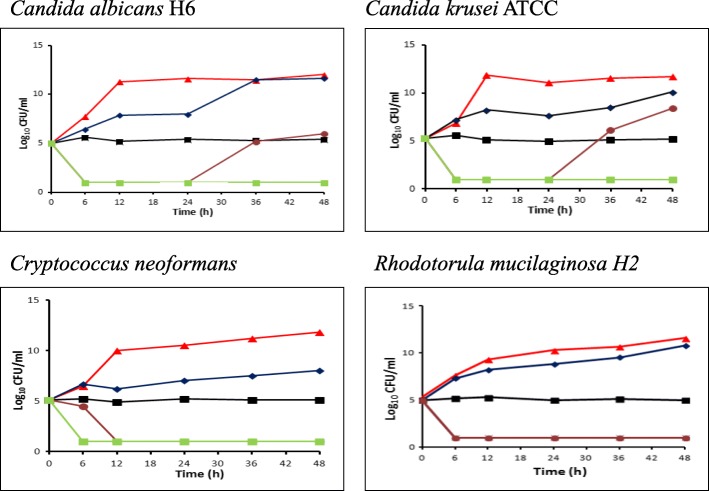
Time-kill kinetics of colistin against four fungal strains (*C. albicans*, *C. krusei*, *C. neoformans* and *R. mucilaginosa*). The colistin concentrations used are as following: (▲) control (no colistin added), (♦) 0.5X MIC, (■) 1X MIC,(●) 2X MIC, (■) 4X MIC

The colistin MICs ranged from 16 to 128 μg/ml for the yeast isolates. As shown on Fig. 1, no inhibitory effect was observed at colistin concentrations equal to 0.5X MICs and the curves were nearly identical to those found in the controls for all species tested. At colistin concentrations equal to 1X MICs, fungistatic effect was observed (Fig. 1). In contrast, colistin fungicidal activities against *C. albicans*, *C. neoformans and R. mucilaginosa* were noted, no later than 12th hour of incubation, with MFC ≥ to two-fold of its MIC values (Fig. 1). For *C. albicans* strains, the MFC was 256 μg/ml, but regrowth was observed at the 36th hour of incubation. The latter phenomenon was not observed in *Cryptococcus neoformans* or in *Rhodotorula mucilaginosa* species.

**Fig. 2:**
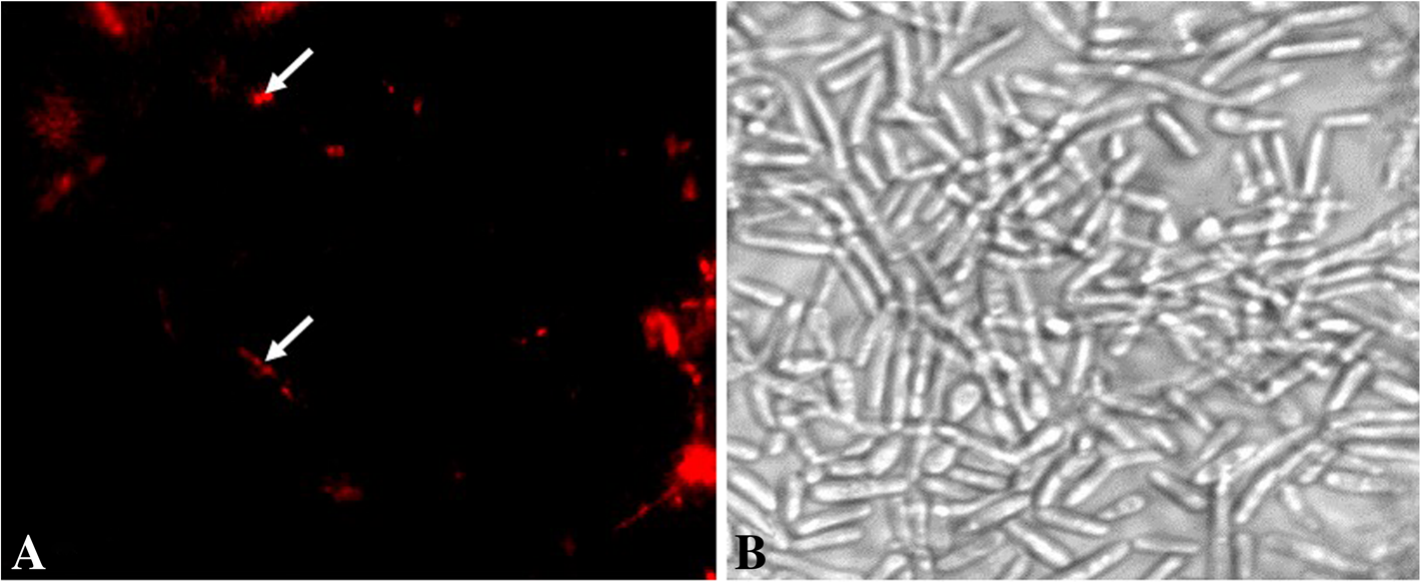
Fluorescence microscopy of *Candida albicans* H6 after treatment with 5 μg/ml of propidium iodide. **a**: fluorescence image of cells treated with 256 μg/ml (2X MIC) of colistin for 24 h. **b**: Brightfield image of cells treated with colistin for 24 h. Scale bar: 2 μm

Moreover, a red fluorescence was observed by fluorescence microscopy (Fig. 2) indicating the presence of interaction between PI and nuclear DNA of *C. albicans* H6 treated with a colistin concentration equal to MFC. This observation allowed us to conclude that colistin can induce cell membrane damage which provide further evidence that it can lead to cell death, confirming its fungicidal activity.

### Synergistic activity of colistin with itraconazole, amphotericin B and fluconazole

**Fig. 3:**
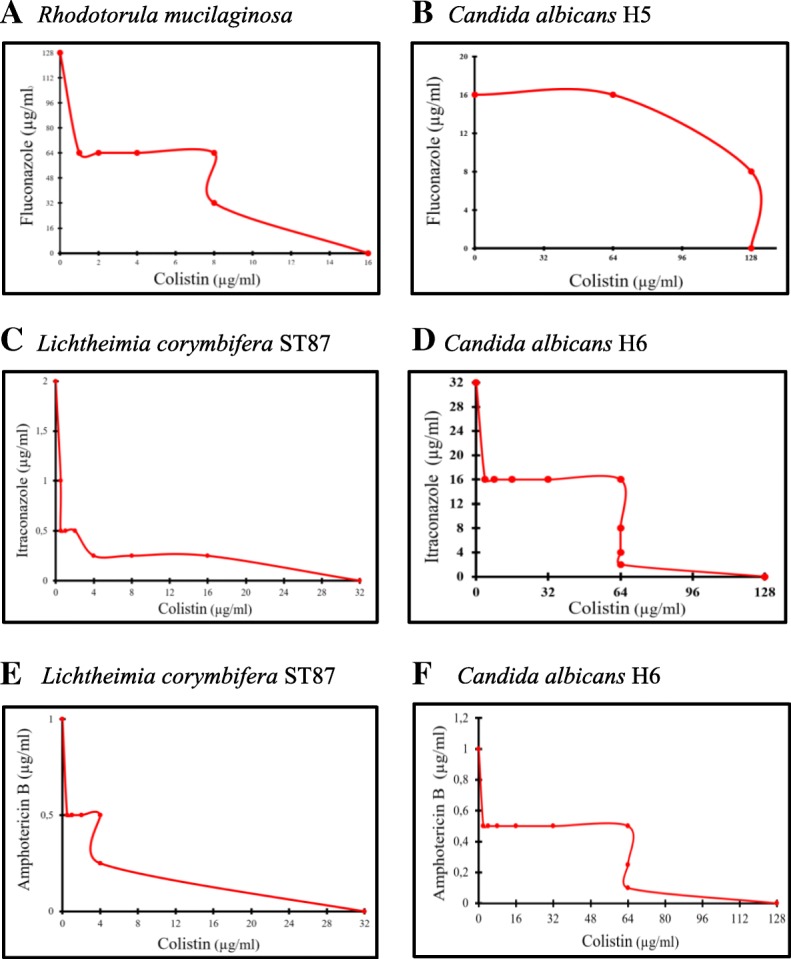
Plots of the checkerboard assays for the combinations of colistin with 3 antifungals (fluconazole, itraconazole and amphotericin **b**). Each dot presents the MICs of colistin (x-axis) and the antifungal agent (y-axis) used in the combination against *R. mucilaginosa* (**a**), *C. albicans* H5 (**b**), *L. corymbifera* (**c** and **e**) and *C. albicans* H6 (**d** and **f**)

Based on checkerboard association testing (Fig. 3), colistin-itraconazole and colistin-amphotericin B exhibited a synergistic activity against MDR *C. albicans* and the mucoralean *Lichtheimia corymbifera.* We noted that itraconazole MIC decreased from 32 μg/ml to 2 μg/ml (for *C. albicans* H6), with a FIX = 0.5, and from 2 μg/ml to 0.5 μg/ml (for *L. corymbifera*), with a FIX = 0.2 when combined with colistin (64 μg/ml and 0.5 μg/ml, respectively) (Table 2). The MIC of amphotericin B decreased from 2 μg/ml to 0.5 μg/ml (for *L. corymbifera*) and from 1 μg/ml to 0.5 μg/ml (for *C. albicans* H6) when combined with colistin (0.5 μg/ml and 1 μg/ml, respectively) (Table 2). Interestingly, colistin acted in synergy with fluconazole against *R. mucilaginosa* but not against *Candida albicans* H5 (Table 2). Although the colistin MICs decreased from 128 μg/ml to 1 μg/ml when combined with fluconazole (16 μg/ml), no synergy against *C. albicans* H5 was observed with a FIX = 1 (Table 2).

**Table 2:** MIC and FIX values of colistin in combination with antifungals from the checkerboard assay

Strains tested	Agents in combination	MIC alone (μg/ml)	MIC in the combination (μg/ml)	FIC	FIX	Outcome
*Rhodotorula mucilaginosa*	Colistin	16	1	0.06	0.5	Synergy
	Fluconazole	128	64	0.5		
*Candida albicans* H5	Colistin	128	1	0.007	1	Additivity
	Fluconazole	16	16	1		
*Candida albicans* H6	Colistin	128	64	0.5	0.5	Synergy
	Itraconazole	32	2	0.06		
	Colistin	128	1	0.007	0.5	Synergy
	Amphotericin B	1	0.5	0.5		
*Lichtheimia corymbifera* ST87	Colistin	32	0.5	0.01	0.2	Synergy
	Itraconazole	2	0.5	0.25		
	Colistin	32	0.5	0.01	0.2	Synergy
	Amphotericin B	2	0.5	0.25		

FIC; Fractional Inhibitory concentration = MIC of the agent in the combination/MIC of the agent alone. FIX; Fractional Inhibitory Index is the sum of the FICs of the agents in the combination

## Discussion

Our study demonstrated that polymyxin antibiotics have, aside from their antibactericidal activity, an antifungal activity, especially against multidrug-resistant *Candida*, *Rhodotorula* and *Cryptococcus* yeast isolates, but also against resistant filamentous fungi, such as *Scedosporium, Rhizopus* and *Lichtheimia* species. The MICs obtained against *Candida* spp. ranged between 64 and 128 μg/ml for colistin and mainly between 16 and 64 μg/ml for PMB. The latter is in concordance with PMB MICs already reported by Zeidler et al and Zhai et al studies [7, 24] confirming the validity of our results and the reproducibility of the used technique. Although we obtained a MIC of PMB equal to 16 μg/ml against *C. neoformans*, a lower MIC (MIC = 8 μg/ml) against this species has been previously described [24]. This could possibly be explained by the resistance phenotype of the strain tested in our study. Moreover, colistin has not been shown, in the early 1970s, to be an effective molecule against three strains belonging to the genus *Rhodotorula* [14]. However, to the best of our knowledge, neither colistin MICs against *C. neoformans* nor PMB MICs against *R. mucilaginosa* have been reported elsewhere.

On the other hand, the filamentous fungi isolates tested in this study constitute the most common emerging cause of human mould infections with an increase being reported from various geographical sites [2], including particularly *Aspergillus* and *Fusarium* spp. Here, colistin and PMB MICs ranging from 16 to 64 μg/ml have been observed against the *F. oxysporum* and *F. Solani* strains which are resistant to almost all azole antifungals and amphotericin B. The same range of MICs has been reported in previous study where 12 *Fusarium* spp. were tested against PMB but not against colistin [16]. The absence of colistin and PMB efficacy against several *Aspergillus* spp. has been reported in various studies with MICs > 256 μg/ml [24, 25] which are similar to our results. Despite that *Aspergillus* spp. are remaining the first cause of mould infections, mucormycosis is increasingly reported in immune-compromised patients and is associated with an elevated rate of mortality (40–70%) even under an appropriate therapy [26]. Among mucoralean pathogens, *Rhizopus* is the main frequently identified genus in human infections. In our study, colistin and PMB MICs against *R. oryzae* are one to two folds higher than those reported in earlier studies [15, 24]. Indeed, in Ben-Ami et al study, the colistin MICs against the fourteen clinical *Rhizopus* spp. tested were variables and ranged between 16 and 32 μg/ml, but MICs of antifungal agents were not mentioned [15]. So the discordance of MIC results between the Ben-Ami et al study and our work could be explained by the eventual high resistance level of our strain to azole agents which can be due to the over expression of efflux-pumps and/or other mechanisms [27]. It is important to mention that PMB MIC against *R. oryzae* was equal to 64 μg/ml in this study compared to 32 μg/ml obtained by Zhai et al study [24].

Finally, among the pertinent emerging fungal pathogens shown by several studies, *Scedosporium* and *Lomentospora* spp. are often notified [28]. They can induce a broad range of diseases; from colonisation in cystic fibrosis patients (for *Scedosporium* spp.) to disseminated severe infections in immuno-compromised hosts (for *Lomentospora prolificans*). Although, the colistin MIC against *S. apiospermum* strain tested here is within the colistin MICs range previously described by Schemuth et al*,* the colistin MIC obtained for *L. prolificans* was higher than that described in this previous study (32 μg/ml versus 12 μg/ml) [29]. Nevertheless, it is worthy to note that MICs_90_ were used by Schemuth et al [29] whereas MICs_100_ were used in our study. Finally, to the best of our knowledge, colistin and PMB activities against *Lichtheimia corymbifera* have not been previously reported.

In human studies, a single dose of 75 to 150 mg of colistin produced bioactive serum colistin concentrations ranging from 6 to 18 μg/ml; higher serum colistin concentrations (13 to 32 μg/ml) were measured during the prolonged therapy of patients with cystic fibrosis [15]. Therefore, the obtained MICs of colistin and PMB are difficult to be achieved with IV administration, mainly due to their renal and neurological toxicities and the risk of frequent selection of bacterial resistant strains.

However, the efficacy of polymyxin molecules on a large number of MDR fungi can be considered advantageous to treat bacterial and fungal co-infections that occur frequently in immunocompromised patients [30] and cystic fibrosis (CF) patients. Chronic bacterial and fungal colonization of the respiratory tract secretions is the main cause of morbidity and mortality in CF patients. Therefore, it would be helpful to use a treatment that is active on both bacteria and fungi in this context. It is worthy to note that, in clinical practice, colistin is administered by inhalation in CF patients as prophylaxis and also as a treatment against *Pseudomonas aeruginosa* infection [31]. In addition, aerosolised colistin treatment, is used in ventilator-associated pneumonia (VAP) cases caused by MDR bacteria in intensive care unit setting [32]. Interestingly, in a recent in vivo study, Landersdorfer et al [33] observed high epithelial lining fluid and low plasma colistin concentrations following the administration of only a pulmonary dose through jet nebulization, confirming a benefit of the local administration of colistin in comparison to its IV treatment [34]. Moreover, a prospective study conducted on 18 patients with chronic lung disease showed that nebulized colistin is effective and improves the quality of life, without presenting side effects and without selecting colistin-resistant isolates in treated patients [35]. So high-dose nebulized colistin could be proposed against pulmonary life-threatening MDR fungi, without increasing colistin plasma concentration, and thus avoiding colistin’s toxicity.

Similar to CF cases, the use of polymyxin antibiotics can improve the poor prognosis of fungal keratitis, due to the emergence of MDR fungal pathogens, particularly *Fusarium* spp. [36], and to the limited ocular penetration of antifungals [37]. Notably, PMB can be formulated for ophthalmic use [16], which is described as a highly effective drug on bacterial corneal ulcerations [38]. Moreover, the use of such antimicrobial agent constitutes a potential alternative treatment that may improve the outcome in some critical infections caused by MDR fungi, such as the recent MDR *Fusarium* keratitis-case report in a 46-year-old man who was still declining even the maximal therapeutic support and therapeutic keratoplasty [36].

Several approaches could be used to overcome the development of antifungal resistance in the treatment of fungal diseases. Aside from the discovery of new effective agents, one realistic alternative option would be to enhance the activity of existing agents. Combination therapies exploit the chances for better efficacy, decreased toxicity and reduced development of drug resistance [39]. A previous study demonstrated an in vitro synergy between colistin and echinocandins in several pathogenic yeasts, namely *C. albicans*, *C. glabrata*, *C. tropicalis*, *C. parapsilosis* and *C. krusei*, as well as in fluconazole-resistant *C. albicans* strains [7].

To the best of our knowledge, no previous studies have tested the activity of colistin in combination with other antifungal agents against fungi of the genera *Rhodotorula* and *Lichtheimia*.

A high decrease of colistin’s MICs was observed when it was combined with azoles (with fluconazole against *R. mucilaginosa* and with itraconazole against either *C. albicans* or *L. corymbifera,* Table 2 and Fig. 3). It is well known that the main mechanism of action of azoles is the inhibition of enzymes that transform lanosterol into ergosterol, a major lipid of the fungal membrane. This inhibition alters both the permeability and fluidity of fungal membrane [40]. On the other hand, polymyxins are well known for weakening the outer membrane in Gram-negative bacteria and the disruption of its permeability leading to a leakage of intracellular components [41]. Therefore, and as supported by PI staining results (Fig. 2), it is likely that antifungal azoles ease the polymyxins’ action and add a potential damage to the fungal membrane which results in a synergistic potency of the combined drugs. Moreover, colistin MIC values significantly decreased from 128 μg/ml to 1 μg/ml and from 32 μg/ml to 0.5 μg/ml against *C. albicans* and *L. corymbifera* respectively when it was associated with amphotericin B. The association of the fungal membrane permeabilization induced by amphotericin B via ion channel formation [42] with the probable membrane damage occurred by colistin could explain the decrease of MICs and the synergistic effect between colistin and amphotericin B.

Thus, despite the elevated MICs of colistin found in our work against multidrug-resistant yeast and moulds, the use of colistin, in combination with other antifungal agents, remains an excellent way to avoid the development of fungal resistance and to decrease the antifungal effective concentration usually used in clinical settings [16, 22].

Colistin is one of many AMPs already used in clinical settings [11]. So, in addition to the colistin-antifungal combination evaluated in this study, other AMPs could further be tested to potentiate the antifungal activity of existing antifungal compounds. For example, Wakabayashi et al, previously described the synergistic effect of lactoferin, a human antimicrobial peptide, with clotrimazole against *C. albicans* [43]. Moreover, lactoferin induced an important decrease of all azoles’ MICs tested against azole-resistant *Candida* spp. [43]. Consequently, natural or synthetic AMPs, have been identified as an original therapeutic alternative that could be investigated by medical researchers and pharmaceutical companies. Using the same approach which was used herein, another AMP, less toxic than polymyxins such as bacitracin or gramicidin analogues, could be tested as monotherapy or in association with antifungals against MDR fungi.

## Conclusion

Our findings demonstrate that polymyxins display a broad-spectrum activity against common MDR fungi especially those which are difficult to manage in clinical settings. Unfortunately, polymyxins’ MICs against these MDR strains are higher than those that could clinically be used in human therapy, thus the use of such high toxicity-associated concentration of polymyxins presents the major limitation of their application in clinical mycology practice. However, colistin seems to induce *C. albicans* membrane damages and to act in synergy with either itraconazole or amphotericin B (each also acting on the fungal membrane). We therefore suggest that colistin (at a ‘safe’ reduced dose) can be used in combination with currently available antifungal drugs, as a last resort option, against life-threatening MDR fungi.

## Abbreviations

AMPs: Antimicrobial peptides
CF: Cystic fibrosis
CLSI: Clinical and Laboratory Standards Institute
FIC: Fractional inhibitory concentration
FIX: Fractional inhibitory index
MDR: Multidrug resistant
MFC: Minimal Fungicidal Concentration
MIC: Minimum inhibitory concentration
PBS: Phosphate-buffered saline
PI: Propidium iodide
PMB: Polymyxin B
